# Aberrant expression of *NPPB* through YAP1 and TAZ activation in mesothelioma with Hippo pathway gene alterations

**DOI:** 10.1002/cam4.6056

**Published:** 2023-05-11

**Authors:** Tatsuhiro Sato, Ken Akao, Ayuko Sato, Tohru Tsujimura, Satomi Mukai, Yoshitaka Sekido

**Affiliations:** ^1^ Division of Cancer Biology Aichi Cancer Center Research Institute Nagoya Japan; ^2^ Department of Molecular Pathology, School of Medicine Hyogo Medical University Hyogo Japan; ^3^ Division of Molecular and Cellular Oncology Nagoya University Graduate School of Medicine Nagoya Japan

**Keywords:** BNP, Hippo pathway, mesothelioma, *NPPB*

## Abstract

**Background:**

Mesothelioma is a neoplastic disease associated with asbestos exposure. It is highly malignant and has a poor prognosis; thus, early detection is desirable. Recent whole‐genome analysis has revealed that mesothelioma is characterized by a high frequency of mutations in a set of genes involved in the Hippo pathway, such as *NF2* and *LATS2*. However, a rapid, simple, and precise method for finding mesothelioma with these mutations has not yet been established.

**Methods:**

Clustering of Hippo pathway gene alteration groups and the differential expression of each gene in mesothelioma patients were analyzed using The Cancer Genome Atlas database. Gene expression levels in various tumors and normal tissues were analyzed using public databases. Knockdown or transient expression of *YAP1* or *TAZ* was performed to evaluate the regulation of gene expression by these genes. NT‐proBNP was measured in the pleural effusions of 18 patients and was compared with NF2 expression in five cases where cell lines had been successfully established.

**Results:**

*NPPB* mRNA expression was markedly higher in the group of mesothelioma patients with Hippo pathway gene mutations than in the group without them. *NPPB* expression was low in all normal tissues except heart, and was highest in mesothelioma. Mesothelioma patients in the high *NPPB* expression group had a significantly worse prognosis than those in the low *NPPB* expression group. *NPPB* expression was suppressed by knockdown of *YAP1* or *TAZ*. NT‐proBNP was abundant in the effusions of mesothelioma patients and was particularly high in those with impaired NF2 expression.

**Conclusions:**

*NPPB*, whose levels can be measured in pleural effusions of mesothelioma patients, has the potential to act as a biomarker to detect *NF2*‐Hippo pathway gene alterations and/or predict patient prognosis. Additionally, it may provide useful molecular insights for a better understanding of mesothelioma pathogenesis and for the development of novel therapies.

## 1 INTRODUCTION

Mesothelioma is a highly aggressive tumor arising from the surface of serous membranes, such as the pleura, peritoneum, and pericardium. Asbestos exposure is a major risk factor for its development. Patients at advanced stages of the disease have a very poor prognosis, with a mean survival of about 12 months, even after cisplatin/pemetrexed combination therapy.^1^ Recent dual therapy with immune checkpoint inhibitors has also shown promise for improving patient outcomes,^2^ however this is not yet conclusive. Although the use of asbestos has already been banned in many countries, including Japan, it is still used in developing countries, and in some countries there are only restrictions on its use, not bans.^3^ Mesothelioma diagnosis utilizes immunohistochemical detection of mesothelioma‐related markers, such as WT‐1 and calretinin, in combination with cancer‐related markers, such as CEA.^3^, ^4^ However, for anatomical reasons, and in the absence of specific clinical manifestations of mesothelioma, patients are often diagnosed only at advanced stages.

Genome‐wide analyses have identified *CDKN2A*, *BAP1*, and *NF2* as genes frequently mutated in mesothelioma.^5^, ^6^ Recent studies have reported that the detection of deletions at the 9p21 locus, where *CDKN2A* is located, by fluorescence in situ hybridization (FISH) and the detection of defective BAP1 expression by immunohistochemical staining can provide a diagnosis of mesothelioma with very high specificity.^4^, ^7^, ^8^, ^9^, ^10^ However, these techniques have not been fully proved for detecting *NF2* gene mutations and protein expression defects.^11^

*NF2* functions as a tumor suppressor by positively regulating the Hippo signaling pathway.^12^, ^13^, ^14^, ^15^ In addition to *NF2*, genetic alterations in *LATS2*, which encodes a component of the Hippo pathway, have been detected in mesothelioma, strongly supporting the idea that disruption of the Hippo pathway induces the development of mesothelioma.^12^, ^16^ Molecularly, the Hippo pathway plays a role in promoting the nuclear export and degradation of the transcriptional coactivators YAP1 and TAZ, whereas disruption of the Hippo pathway increases the nuclear accumulation of these proteins. YAP1 and TAZ in the nucleus bind to TEAD family DNA‐binding proteins (TEAD1–4) and activate the transcription of tumor‐promoting genes. Previous studies have demonstrated that shRNA‐mediated silencing of YAP1 or TAZ strongly suppresses cell growth in mesothelioma cells with *NF2* mutations, while expression of activating mutants of YAP1 or TAZ promotes tumorigenesis in normal immortalized mesothelial cells.^17^, ^18^, ^19^ In recent years, several TEAD inhibitors that block YAP1 signaling have been successively developed.^20^, ^21^, ^22^ Experiments using mouse models have reported that TEAD inhibitors dramatically inhibit the growth of mesothelioma cells with Hippo pathway gene mutations.^20^ Given this situation, it is necessary to develop easier and more reliable methods to assess Hippo pathway inactivation and YAP1 and TAZ activation in actual patient pleural fluid and tumor samples in order to appropriately administer these new drugs to mesothelioma patients.

In this study, we identified *NPPB* as a gene that was significantly upregulated in the samples from mesothelioma patients with Hippo pathway alterations. The transcript of *NPPB* is the brain natriuretic peptide (BNP) precursor, proBNP, which is further cleaved to produce BNP and NT‐proBNP. The *NPPB* gene product is known as BNP, which is one of the natriuretic peptides (NPs), including atrial NP (ANP, *NPPA* gene product) and C‐type NPs (CNP, *NPPC* gene product).^23^, ^24^, ^25^, ^26^ There are three known receptors for NP: NP receptor A (NPR‐A, *NPR1* gene product), NP receptor B (NPR‐B, *NPR2* gene product), and NP receptor C (NPR‐C, *NPR3* gene product). BNP binds to NPR‐A, but not NPR‐B, and regulates various intracellular signals to exert its biological effects. NPR‐C can bind to multiple NPs including BNP and functions as a clearance receptor.^27^, ^28^ NT‐proBNP can be used as an alternative to BNP for measuring blood levels because it has no physiological activity but has a long half‐life. BNP and NT‐proBNP play a central role in the regulation of heart failure and are biomarkers that can be used to assess the severity of heart failure.^29^, ^30^, ^31^ They have also been found useful in the diagnosis and prognosis of acute coronary syndromes and stable ischemic heart disease.^32^, ^33^ Although high levels of BNP have been reported in pleural effusions from mesothelioma patients,^34^ little is known about why BNP is highly expressed and how it relates to clinical characteristics.

## 2 METHODS

### 2.1 Cell lines and pleural fluids

HOMC‐D4 cell lines established in our laboratory^35^ were used for assays at passages 10–20. They were deposited and are now available from RIKEN BioResource Center. NCI‐H2052 and NCI‐H2373 cells were purchased from the American Type Culture Collection. All cell lines were cultured in RPMI‐1640 medium supplemented with 10% (v/v) fetal bovine serum (FBS) and 1% (v/v) antimicrobials at 37°C in a humidified incubator with 5% CO_2_. Pleural fluid samples from patients were collected and centrifuged at 3000 rpm for 10 min, and the supernatant was stored at −80°C until use. The research protocol was approved by the Ethics Committee of Aichi Cancer Center Research Institute.

### 2.2 Antibodies and reagents

Anti‐YAP1 (#14074), anti‐phospho‐YAP1 (Ser127) (#13008), anti‐TAZ (#4883), and anti‐TEAD1 (#12292) antibodies were purchased from Cell Signaling Technology. FLAG‐M2 and anti‐β‐actin (#A5441) antibodies were sourced from Sigma. K‐975 was synthesized and purified to >99% purity by Namiki Shoji Co. Ltd.

### 2.3 Public databases

The mRNA and protein expression levels and clinical data of patients with mesothelioma and other cancers were analyzed from The Cancer Genome Atlas (TCGA) dataset on the cBioportal website (https://www.cbioportal.org/). The mRNA expression levels of various cancer cell lines were analyzed from DepMap Public 22Q1 dataset from DepMap portal website (https://depmap.org/portal/), and the *NPPB* mRNA expression levels of each normal organ were analyzed from GTEx Analysis Release V8 in the GTEx data portal website (https://gtexportal.org/home/).

### 2.4 qRT‐PCR

Total RNA was prepared using an RNeasy Plus RNA extraction kit (Qiagen) according to the manufacturer's protocol. Random‐primed, first‐strand cDNA was synthesized from total RNA using the ReverTra Ace qPCR RT Kit (Toyobo). qRT‐PCR analyses were performed in triplicate using the KAPA SYBR Fast qPCR kit (KAPA Biosystems) in the QuantStudio 3 system (Applied Biosystems). Relative gene expression normalized to the expression of β‐actin as a reference gene was calculated using the ΔΔCt method with efficiency correction. The primer sequences used in this study were as follows: β‐actin, 5′‐CCAACCGCGAGAAGATGA‐3′ and 5′‐CCAGAGGCGTACAGGGATAG‐3′; *NPPB*, 5′‐TCTGGCTGCTTTGGGAGGAAGA‐3′ and 5′‐CC TTGTGGAATCAGAAGCAGGTG‐3′; *ANKRD1*, 5′‐GTGG AAAAGCGAGAAACAACGA‐3′ and 5′‐TCAGGTTCTGG TTCCTTTACAACT‐3′.

### 2.5 Western blotting

Cells were lysed with 1× SDS sample buffer (3% (w/v) SDS, 5% (v/v) glycerol, 62 mM Tris–HCl [pH 6.8], 5% (v/v) 2‐mercaptoethanol) and incubated at 95°C for 5 min. The proteins were resolved by a 5%–12% gradient polyacrylamide gel (#192‐15201; Fujifilm), transferred onto a polyvinylidene fluoride membrane (#IPVH00010; Merck) and probed with a 1000‐fold dilution of antibodies and horseradish peroxidase‐conjugated secondary antibodies (Cell Signaling Technologies) in 5% (w/v) skim milk. Protein bands were detected using ECL Prime Detection Reagent (#RPN2236; GE Healthcare) and Amersham Imager 680 (GE Healthcare).

### 2.6 Enzyme‐linked immunosorbent assay (ELISA)

Cell culture medium was collected 48 h after seeding the cells. The amount of BNP secreted from the cells into the culture medium was quantified using a Human BNP ELISA Kit (#ab193694, Abcam) according to the manufacturer's instructions. The absorbance was measured using SpectraMax iD3 (Molecular Devices LLC).

### 2.7 NT‐proBNP measurement

The amount of NT‐proBNP in the supernatant of the pleural fluid was measured by a clinical laboratory (BML Inc.).

### 2.8 Statistical analysis

Comparisons between two groups of data obtained via qRT‐PCR or ELISA analysis were performed using Student's *t*‐test. The experiments were repeated at least three times. Statistical analysis of TCGA clinical data were performed using GraphPad Prism 9.

## 3 RESULTS

### 3.1 Characteristics of mesothelioma with mutations in the Hippo pathway gene

**FIGURE 1:**
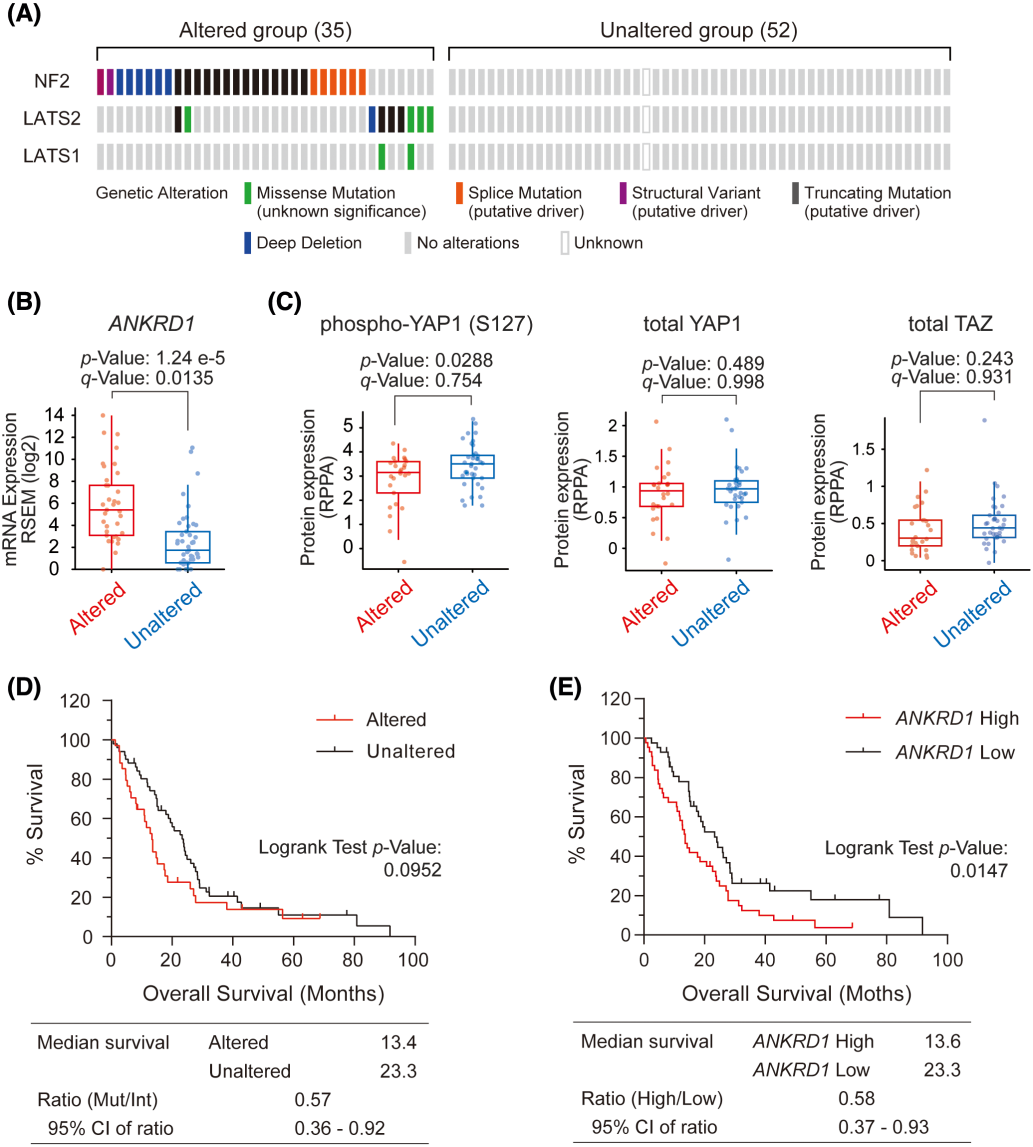
Characteristics of the Hippo pathway gene alteration group in The Cancer Genome Atlas‐mesothelioma cohort. (A) Oncoprint of genetic alterations in 87 patients with mesothelioma; the group of patients with alterations in either *NF2*, *LATS1*, or *LATS2* was classified as Altered group, and the other group as Unaltered group. In parentheses, the number of patients. Color differences indicate different types of genetic alterations. (B, C) Box plot showing gene expression levels of *ANKRD1* (B), protein phosphorylation of YAP1 at serine 127 and total YAP1 protein expression levels (C) in Altered and Unaltered groups. (D, E) Kaplan–Meier curves of overall survival in Altered and Unaltered groups (D) or in *ANKRD1* high‐expressing and low‐expressing groups (E).

We first examined the clinical characteristics of mesothelioma with mutations in the Hippo pathway genes using data from 87 samples from the TCGA mesothelioma (TCGA‐MESO) cohort. Since mutations in the *NF2*, *LATS1*, and *LATS2* genes are relatively frequent among all the genes related to the Hippo pathway in mesothelioma,^6^ patients with genetic alterations such as mutations, deletions, or gene fusions in these genes were designated as the Altered group (Hippo pathway gene alteration group) and analyzed in comparison with the other group (Unaltered group). 43% (35/82) of the patients were classified as the Altered group and had no significant differences with the other group in terms of cancer progression, sex, or total number of mutations (Figure 1A). Besides, increased *ANKRD1*, a well‐known gene downstream of YAP1, was detected in the Altered group (Figure 1B). Alterations in Hippo pathway genes are known to increase the amount of unphosphorylated YAP. Still, the results of our analysis did not reach statistical significance despite a trend toward a decrease in phosphorylated YAP in samples with alterations in Hippo pathway genes (*p* = 0.0288, *q* = 0.754). YAP1 and TAZ expression levels were not significantly different between the two groups, and there were no available data on phosphorylated TAZ (Figure 1C). Regarding patient prognosis, the median overall survival (OS) of the Altered and Unaltered groups was 13.4 and 23.3 months, respectively (Figure 1D). There was no significant difference (*p* = 0.0952) in OS between the two groups; still, there was a trend toward poorer prognosis in the Altered group. We further compared the OS of patient groups according to the expression levels of ANKRD1. Kaplan–Meier plot analysis results showed that the ANKRD1‐High group had a significantly worse prognosis (*p* = 0.0147) than the ANKRD1‐Low group (Figure 1E). The discrepancy in the results of these survival analyses indicates the difficulty of understanding the effects on the Hippo pathway based on only a few known genetic changes or single gene expression levels.

### 3.2 NPPB is highly expressed in mesothelioma with alterations in Hippo pathway genes

**FIGURE 2:**
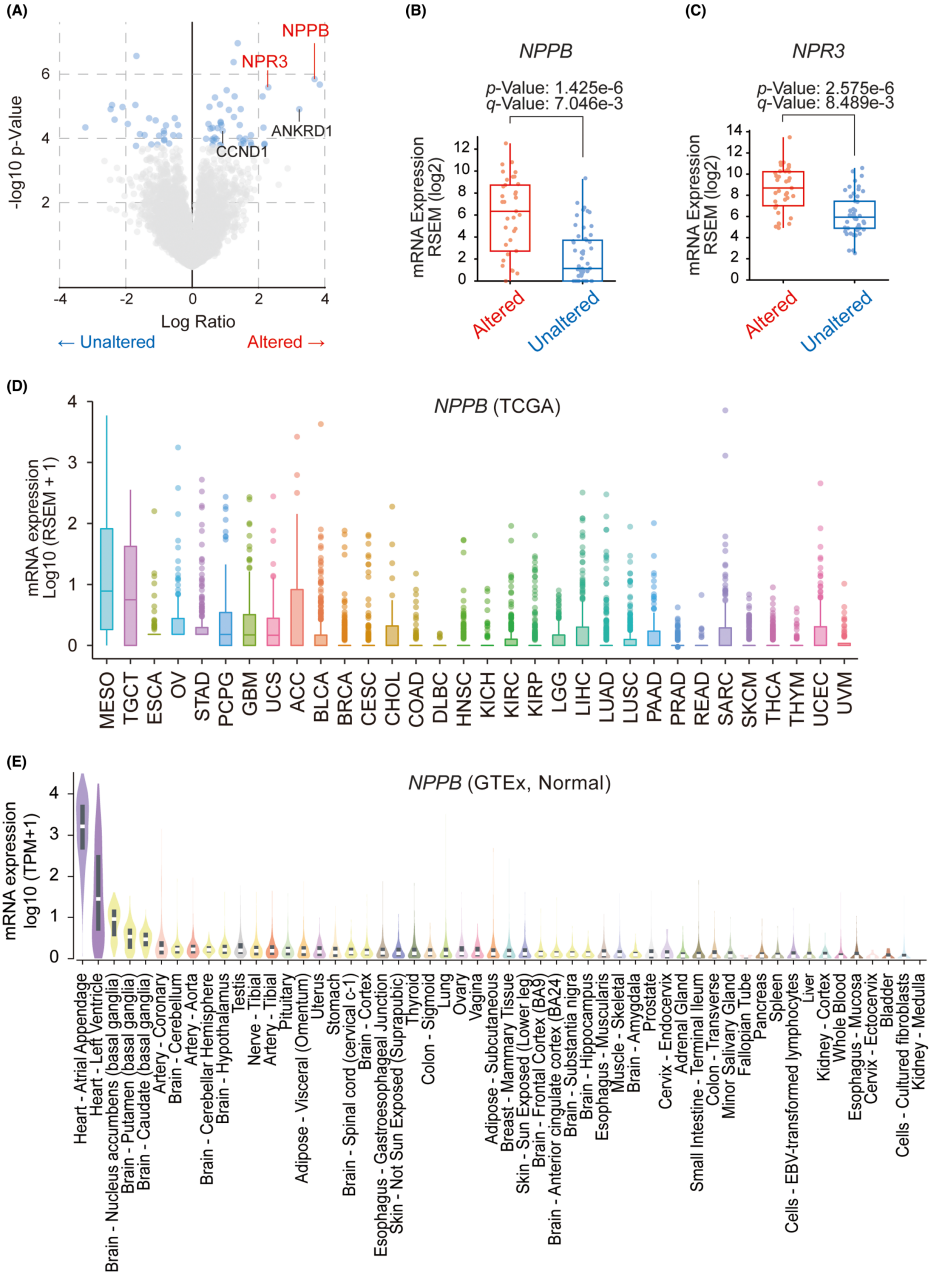
High expression of *NPPB* in mesothelioma patients with Hippo gene alterations. (A) Volcano plot showing differentially expressed genes between Altered and Unaltered groups. Blue dots, genes with *q*‐value >0.05. (B, C) Box plot showing gene expression levels of *NPPB* (B) and *NPR3* (C) in the Altered and Unaltered groups. (D) Box plot showing the expression levels of *NPPB* in 32 cancer types in TCGA database. Dots indicate outliers. (E) Violin plot showing the expression levels of *NPPB* in each of the human tissue types in the GTEx database. Outliers were eliminated.

Using the gene expression data obtained from the RNA‐seq analysis, we further searched for genes whose expression levels differed between the Altered and Unaltered groups. A total of 68 genes were significantly upregulated in the Altered group (Figure 2A, Table S1). These genes included *ANKRD1* and *CCND1*,^19^ which are known to be induced by YAP1 activation. Notably, expression of *NPPB*, which encodes a BNP, a powerful biomarker for heart failure diagnosis, was more than 13‐fold higher in the Altered group (Figure 2A,B). In addition, levels of *NPR3*, which encodes the NP clearance receptor, were 5‐fold higher in the Altered group (Figure 2A,C). No differences in expression levels of genes encoding other NPs or NP receptors were found between the two groups (Figure S1). To investigate whether *NPPB* and *NPR3* are upregulated in other types of cancer besides mesothelioma, we analyzed data from 9279 TCGA tumors across 32 cancer types. *NPPB* expression was strongly suppressed in most cancer types, but strongly expressed in mesothelioma (Figure 2D), while *NPR3* was highly expressed in other cancer types, including medulloblastoma (Figure S2). Consistently, cell lines derived from mesothelioma cell patients had higher *NPPB* expression levels than those derived from other cancer types (Figure S3). Notably, *NPPB* is rarely expressed in any normal tissues except the heart (Figure 2E). Therefore, we conducted further studies focusing on the changes in *NPPB* expression.

### 3.3 Patients with mesothelioma expressing high levels of NPPB have a poor prognosis

**FIGURE 3:**
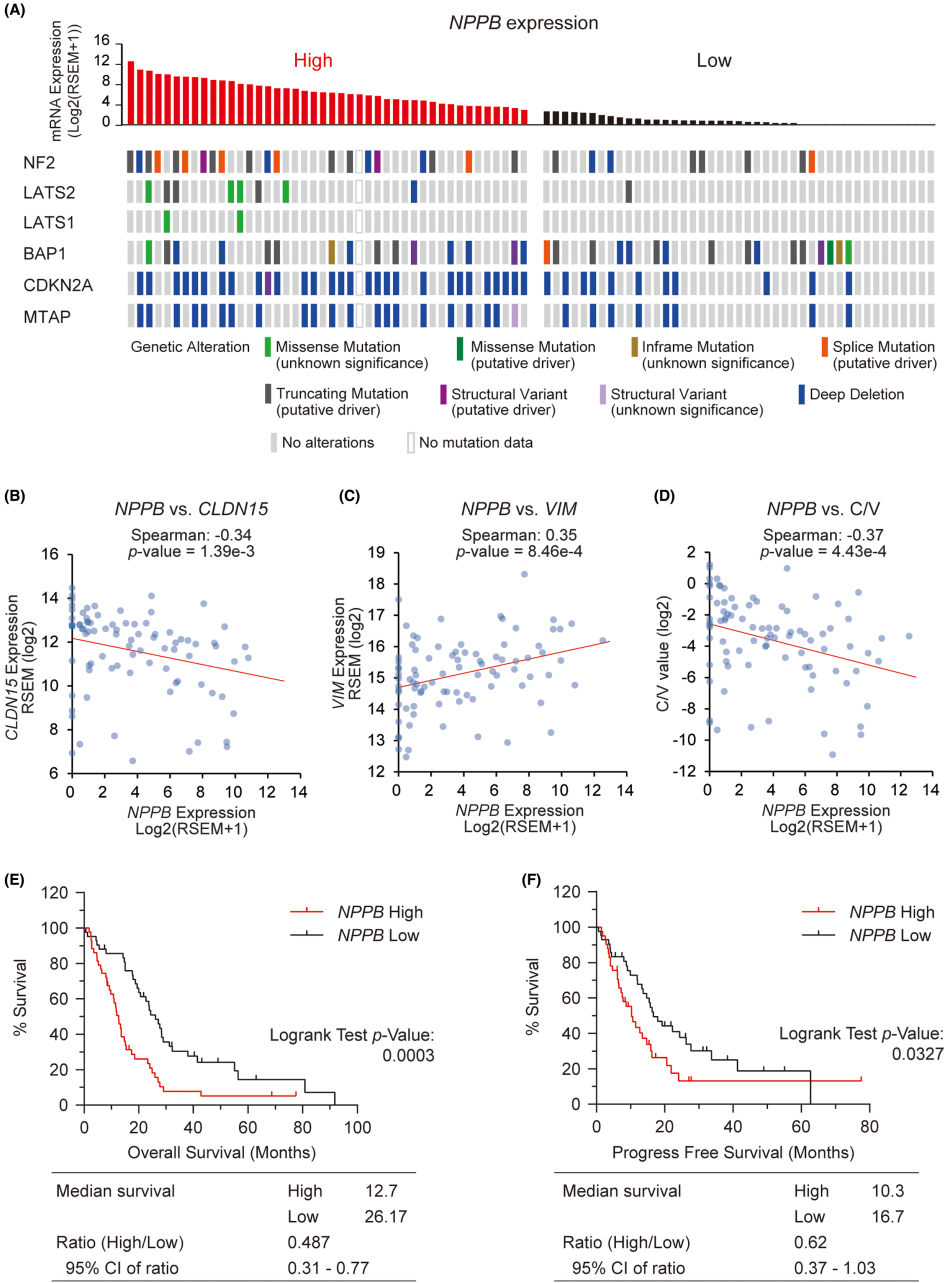
Characteristics of groups divided according to *NPPB* expression levels in The Cancer Genome Atlas‐mesothelioma (TCGA‐MESO) cohort. (A) Oncoprint showing the genetic alterations of the patients in TCGA‐MESO cohort with high and low *NPPB* expression. Color differences indicate different types of genetic alterations. (B–D) Dot plots showing the correlation between *NPPB* and *CLDN15* (B), *VIM* (C), or *CLDN15/VIM* (D) (C/V) gene expression values in TCGA‐MESO cohort data. Red lines represent regression lines. (E, F) Kaplan–Meier curves of overall survival (E) and progression‐free survival (F) in *NPPB* High and *NPPB* Low groups.

To gain a better understanding of the characteristics of the high‐*NPPB* group, we divided the patients enrolled in TCGA‐MESO cohort into two groups (High and Low) according to their *NPPB* expression levels (Figure 3A). The genetic data showed that patients with alterations in the Hippo pathway or *CDKN2A* genes were significantly more common in the High group than in the Low group (*p* = 0.0003 and 0.0002, respectively; Table 1). On the other hand, patients with *BAP1* mutations were not statistically different between the High and Low groups.

**TABLE 1:** Clinical characteristics of groups divided according to *NPPB* expression levels in The Cancer Genome Atlas‐mesothelioma cohort.

Characteristics	NPPB expression		*p*‐value
	High (*N* = 44)	Low (*N* = 43)	
Hippo gene mutation			0.0003*
Yes	26 (67%)	9 (33%)	
No	18 (35%)	34 (65%)	
BAP1 gene mutation			0.8248
Yes	15 (48%)	16 (52%)	
No	29 (52%)	27 (48%)	
Sex			0.2909
Female	10 (63%)	6 (38%)	
Male	34 (48%)	37 (52%)	
Histological type			0.3505
Epithelioid	12 (52%)	11 (48%)	
Biphasic	30 (48%)	32 (52%)	
Sarcomatoid	2 (100%)	0 (0%)	
Disease stage			0.8824
I	6 (60%)	4 (40%)	
II	8 (50%)	8 (50%)	
III	23 (51%)	22 (49%)	
IV	7 (44%)	9 (56%)	
Tumor stage			0.4471
T1	8 (57%)	6 (43%)	
T2	16 (62%)	10 (38%)	
T3	15 (47%)	17 (53%)	
T4	4 (31%)	9 (69%)	
TX	1 (50%)	1 (50%)	
Lymph node stage			0.1182
N0	25 (57%)	19 (43%)	
N1	7 (70%)	3 (30%)	
N2	11 (42%)	15 (58%)	
N3	1 (33%)	2 (67%)	
NX	0 (0%)	4 (100%)	
Metastasis stage			0.2183
M0	28 (49%)	29 (51%)	
M1	3 (100%)	0 (0%)	
MX	13 (48%)	14 (52%)	

Hippo gene mutation indicates genetic alterations in one or more of the NF2, LATS1, or LATS2 genes. The *p*‐value for statistically significant term is indicated in bold with an asterisk.

There were no significant differences between the two groups in terms of clinical characteristics, including sex, pathology, and cancer progression (Table 1). Both sarcomatoid tumors in the histological classification were part of the *NPPB* High group, but this showed no statistically significant difference owing to there being too few cases. Remarkably, however, *CLDN15* expression, which is high in epithelioid mesothelioma, was significantly negatively correlated with *NPPB* expression (Figure 3B). Furthermore, *NPPB* expression showed a significant positive correlation with that of *VIM*, which is upregulated in sarcomatoid mesothelioma (Figure 3C). *CLDN15*/*VIM* expression values, which show a gradient in epithelial to sarcomatoid subtypes, also showed a significant negative correlation with *NPPB* expression (Figure 3D). Kaplan–Meier analysis of patient survival showed that the median OS in the Low *NPPB* group was 26.1 months (95% CI, 19.4–31.2) whereas that in the high *NPPB* group was 12.7 months (95% CI, 9.3–15.1; Figure 3E). Similarly, median PFS in the low *NPPB* group was 16.7 months (95% CI, 13.3–26.2) whereas that in the high *NPPB* group was 10.3 months (95% CI, 6.6–14.7) (Figure 3F).

### 3.4 NPPB transcription is promoted by the YAP1‐TEAD complex

We investigated whether high *NPPB* expression has a direct effect on tumor cell proliferation. We found that *NPPB* knockdown did not suppress proliferation in cells with either *NF2* (NCI‐H2052, NCI‐H2373) or *LATS2* (Y‐MESO‐27) gene alterations (Figure S4A). However, gene ontology analysis revealed numerous genes related to cell division and mitotic nuclei expressed differently between the High and Low *NPPB* expression groups (Figure S4B), suggesting that these variations may promote tumor growth.

**FIGURE 4:**
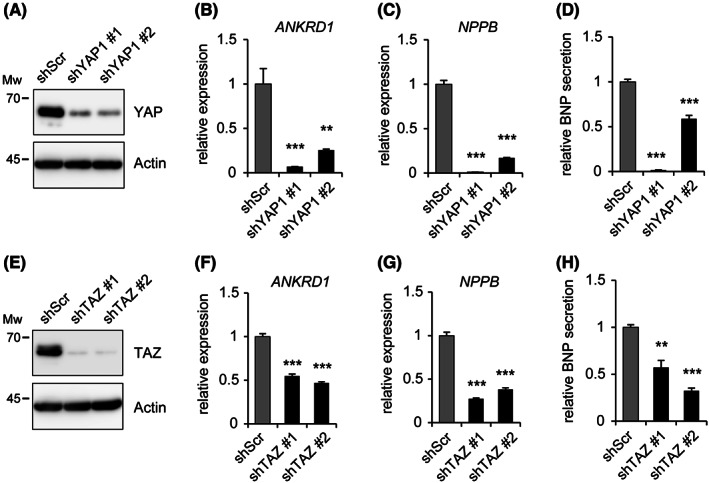
Reduced *NPPB* expression following YAP1 or TAZ knockdown. (A) NCI‐H2373 cells expressing shRNA targeting YAP1 (shYAP1 #1 and #2) or Scramble shRNA (shScr) as a control were generated using lentivirus and subjected to western blotting. (B, C) The expression ratio of *ANKRD1* (B) or *NPPB* (C) in each cell was measured via qRT‐PCR. (D) The culture medium of NCI‐H2373 cells expressing shYAP1 or shScr was subjected to enzyme‐linked immunosorbent assay (ELISA) to assess the amount of secreted brain natriuretic peptide (BNP). (E–H) Using NCI‐H2373 cells expressing shTAZ (shTAZ #1, #2) or shScr, the same experiments as described in A–D were performed.

In mesothelioma, cells with mutations in Hippo pathway genes, including *NF2*, YAP1, and TAZ are constitutively activated to enhance the expression of a series of genes involved in cell proliferation. Therefore, we performed knockdown experiments to determine whether *NPPB* is regulated by YAP1 and/or TAZ. Silencing YAP1 or TAZ expression in NCI‐H2373 cells was performed by expressing shRNA against each gene (shYAP1 or shTAZ), and was confirmed via western blotting (Figure 4A,E). Knockdown of *YAP1* or *TAZ* resulted in decreased *ANKRD1* mRNA expression compared to control cells (Figure 4B,F). *NPPB* expression also decreased in YAP1 and TAZ knockdown cells (Figure 4C,G). Furthermore, BNP secretion in the cell culture medium also decreased in knockdown cells compared to control cells (Figure 4D,H).

**FIGURE 5:**
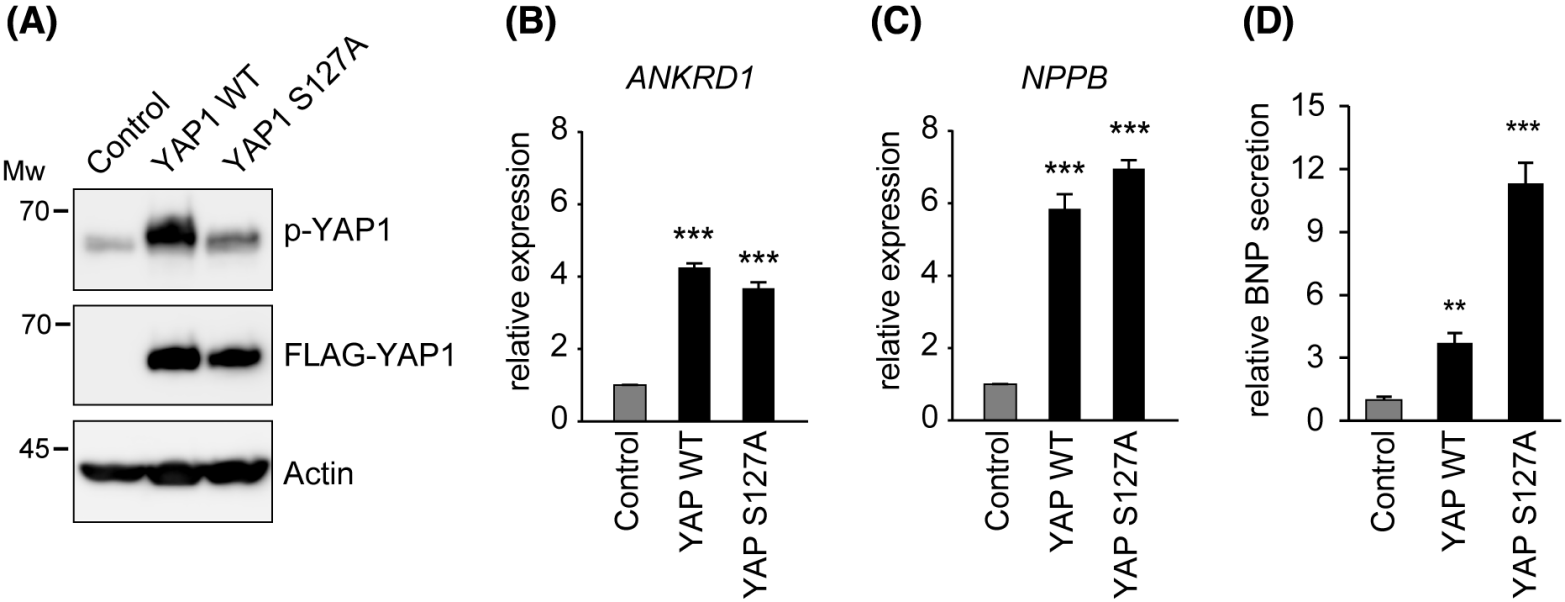
Induction of *NPPB* expression through YAP1 activation. (A) NCI‐H2373 cells or the cells expressing wild‐type YAP1 (YAP1 WT) or constitutively active YAP1 mutant (YAP1 S127A) fused with FLAG tag were generated using lentivirus and subjected to western blotting. (B, C) The expression ratio of *ANKRD1* (B) or *NPPB* (C) in each cell was measured via qRT‐PCR. (D) The culture medium of NCI‐H2373 cells or the cells expressing YAP1 was subjected to enzyme‐linked immunosorbent assay (ELISA) to assess the amount of secreted brain natriuretic peptide (BNP).

To further investigate whether YAP1 activation is sufficient to increase *NPPB* expression, FLAG‐tagged wild‐type YAP1 or an activated YAP1 mutant (YAP1 S127A) was overexpressed in HOMC‐D4 normal immortalized mesothelial cells. Each YAP1 protein was expressed to the same extent, as confirmed with western blotting using FLAG antibody (Figure 5A). Phosphorylated YAP1, an indicator of YAP1 inactivation, was also detected, confirming the activation of the S127A mutant. Wild‐type YAP1 and activated YAP1 mutants significantly increased mRNA levels of both *ANKRD1* and *NPPB* (Figure 5B,C). Also, YAP1 S127A promoted extracellular BNP secretion more strongly than YAP1 WT (Figure 5D). These results indicate that, in mesothelioma cells, *NPPB* expression is regulated by YAP1 and TAZ.

### 3.5 Measurement of NT‐proBNP may be a useful biomarker for mesothelioma treatment

**FIGURE 6:**
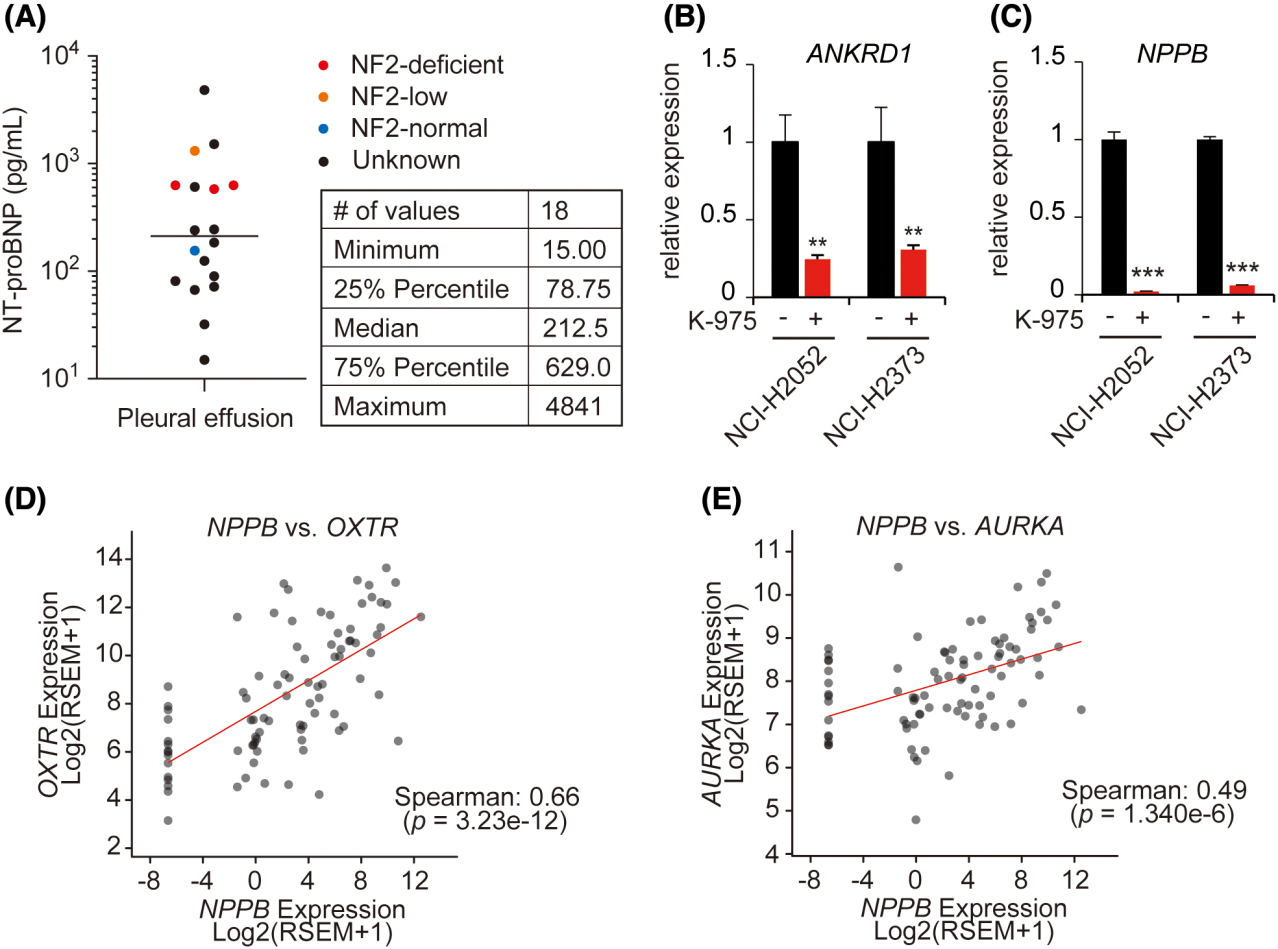
Exploring the clinical value of *NPPB* expression levels. (A) NT‐proBNP levels were measured using pleural fluid collected from 18 patients. The NF2 expression ratios established from the pleural fluid in five cell lines are shown in color. (B, C) After purification of mRNA from cells treated with or without K‐975, the expression ratios of *ANKRD1* (B) and *NPPB* (C) were measured via qRT‐PCR. (D, E) Dot plot showing the expression correlations of the indicated genes using The Cancer Genome Atlas‐mesothelioma cohort data. Red lines represent regression lines.

Since YAP1 and TAZ are potential therapeutic targets, detection of BNP released from mesothelioma cells is expected to be a predictive marker of therapeutic response. Therefore, we measured BNP secretion as NT‐proBNP levels in pleural effusions collected from 18 mesothelioma patients. NT‐proBNP levels were widely distributed as 18–4841 pg/mL, with a median of 212.5 pg/mL (Figure 6A). In addition, five mesothelioma cell lines had been established from these effusions: three with complete NF2 inactivation, one with relative NF2 downregulation with undetermined mechanism, and one with NF2 intact (Figure 6A). In samples with normal NF2 expression, pleural fluid NT‐proBNP levels were below median; and in samples with low or undetectable NF2 expression, levels were above the median. Although statistical tests could not be performed due to the small number of cell lines established, the results suggest that mesotheliomas with Hippo pathway gene mutations may secrete high amounts of NT‐proBNP in accordance with their gene expression levels.

Furthermore, we examined the possibility that increased *NPPB* expression may be a suitable biomarker for tumor detection and evaluation of therapeutic efficacy. The recently developed TEAD inhibitor K‐975 suppresses the transcriptional activity of YAP1 and TAZ and potently inhibits the growth of mesothelioma with Hippo pathway gene mutations both in vitro and in vivo. To examine whether *NPPB* could serve as a marker to monitor the therapeutic response to K‐975, we measured *NPPB* expression in mesothelioma cells before and after K‐975 treatment. The expression of *NPPB* mRNA was more strongly suppressed than that of *ANKRD1* in cells treated with K‐975 (Figure 6B,C), suggesting that monitoring the expression levels of *NPPB* or its gene products may be an indicator of inhibitor efficacy.

Finally, we searched for genes whose expressions correlated with *NPPB* to investigate the potential of *NPPB* for clinical use in mesothelioma patients. Among 19,782 genes in the TCGA‐MESO database, *OXTR* (Spearman's correlation coefficient 0.66, *p* < 0.001) was identified as the gene with the highest positive correlation (Figure 6D). *OXTR* is highly expressed in mesothelioma and reported as a potential therapeutic target.^36^ Among other genes with significant correlations, *AURKA* was also found (Spearman's correlation coefficient 0.49, *p* < 0.001) (Figure 6E). The *AURKA* transcript, aurora kinase, is a known target of the anticancer drug alisertib, which is currently in clinical trials for mesothelioma patients. Since the expression of aurora kinase is strongly correlated with that of *MYC*,^37^ it was suggested that *MYC* copy number could be a predictor of susceptibility to alisertib. However, Phase II clinical trials have recently revealed that *MYC* copy number increase or gene amplification does not correlate with response rate or improved patient outcome.^38^ Our data present the possibility of *NPPB* or NT‐proBNP replacing *MYC* as a new marker to predict sensitivity to aurora kinase inhibitors.

## 4 DISCUSSION

In this study, we found that *NPPB* is most highly expressed in mesothelioma among 32 cancer types, and that its expression is induced in the mesothelioma (Altered) group with *NF2* or *LATS1/2* gene mutations. These results are consistent with previous studies reporting significantly higher BNP levels in pleural fluid from mesothelioma patients than in pleural fluid from other cancer patients such as lung cancer patients.^34^ A previous ChIP analysis using glioblastoma cells suggested transcriptional activation by YAP1 as a molecular mechanism for *NPPB* high expression.^39^ In the present study, we also showed that YAP1 and TAZ enhance *NPPB* transcription in *NF2*‐deficient cells. These findings will shed new light on the development of detection methods and therapeutics for mesothelioma.

It is worth noting that pathway‐based approaches have their limitations: the molecules involved in the Hippo signaling pathway and their regulators are not yet well defined and thus, it is difficult to fully understand the impact of known genetic variants on the pathway. Protein array data may also not fully capture pathway activity due to the limited number of molecules analyzed and incomplete pathway coverage compared to that of other high‐throughput technologies. In this regard, expression of BNP and NT‐proBNP is expected to be a potentially useful additional tool estimating YAP1 and TAZ activation in tumors. Although no effective drugs have yet been developed for mesothelioma patients with Hippo pathway mutations or YAP1/TAZ hyperactivation, a number of pan‐TEAD inhibitors that block YAP1/TAZ activity and compounds that act only on specific TEAD proteins have recently been reported.^20^, ^40^, ^41^ Furthermore, a Phase 1, first‐in‐human clinical trial has been initiated to investigate the efficacy of these agents in mesothelioma patients with *NF2* mutations (ClinicalTrials.gov Identifier: NCT04665206, NCT05228015). Thus, although it will need to be evaluated in future clinical trials, measurement of BNP and NT‐proBNP expression is expected to be a suitable biomarker for predicting the response to these drugs.

It should be noted, that increased expression of BNP and NT‐proBNP is observed in heart failure; however, in the present study, we were unable to collect sufficient clinical information on cardiovascular function from the patients. Previous reports have shown that BNP levels are elevated in plasma and pleural fluid in mesothelioma patients. However, there are differences between mesothelioma and heart failure patients. Specifically, in heart failure patients, BNP levels in plasma are several times higher than in pleural fluid; conversely, in mesothelioma patients, BNP levels are higher in pleural fluid than in plasma.^34^ This difference may be caused by local tumor BNP production in mesothelioma tissue. Still, the biological consequences of BNP entering the blood from the pleural fluid need to be carefully examined in future studies.

Analysis of the TCGA‐MESO dataset in this study revealed for the first time that mesothelioma patients with high *NPPB* expression had a much poorer prognosis than those with low *NPPB* expression. On the other hand, when mesothelioma patients were divided into two groups (with and without Hippo pathway gene alterations), there was no significant difference in prognosis between them. This suggests that *NPPB* may play an important function in tumorigenesis and malignant transformation independently of *NF2* or *LATS1/2* gene alterations. However, our experiments did not confirm that *NPPB* promotes mesothelioma cell proliferation directly. In this regard, it is possible that *NPPB* expression exerts a coordinated effect not only on its own cells but also on surrounding cancer cells. The *NPPB* gene product, BNP, has been reported to exert paracrine effects on the proliferation of surrounding fibroblasts after being secreted out of cells.^42^ Since the tumor microenvironment has important prognostic implications for mesothelioma patients,^43^ the effects of BNP released outside mesothelioma cells on immune cells and peritumoral cells should be investigated in the future. In addition, since the *CDKN2A* mutation in mesothelioma patients is known to show a worse prognosis, *CDKN2A* inactivation may also be associated with *NPPB* expression.

Immunohistochemistry (IHC) is the most common method to detect aberrant expression of a particular protein. However, reliable detection of disturbed NF2 expression in mesothelioma using this technique is still under development.^11^ Instead, the FISH method, which detects gene deletions, has been reported to show good sensitivity and specificity in detecting loss of *NF2* in mesothelioma.^44^ Nevertheless, FISH still has some technical challenges, such as an ambiguous cutoff value used to evaluate the signal. The mechanisms of gene inactivation in mesothelioma are diverse, including genetic and epigenetic events.^45^ Detection of the *NPPB* gene product using IHC may help detect mesothelioma cells that have genetic alterations in Hippo pathway genes, including *NF2* and *LATS1/2*, or that have YAP1 and TAZ activated by these mutations.

It should also be noted that in the present analysis, it is still unclear whether changes in Hippo pathway genes or *NPPB* mRNA expression correlate with the intracellular synthesis or extracellular secretion of NP‐proBNP in pleural effusions. In Figure 6, we show that NT‐proBNP in the original pleural fluid was elevated in four of the five established cell lines with *NF2* alterations, suggesting that *NF2* alterations may result in elevated NT‐proBNP, albeit in a small number of cases. Accordingly, we have purchased several commercial antibodies and tried to quantify BNP via IHC; however, we are yet to find one capable of estimating BNP expression levels. Developing suitable antibodies for IHC and validating their clinical usefulness will be one of the next objectives of our research.

## 5 CONCLUSIONS

We found that *NPPB* transcription and extracellular secretion of its gene products, BNP and NT‐proBNP, were enhanced in mesothelioma patients with Hippo pathway gene alterations. Thus, our findings might contribute to the prediction of the efficacy of new therapeutic strategies that target Hippo signaling alteration. High expression of *NPPB* was also associated with poor patient prognosis. Further studies are needed to determine the impact of *NPPB* on mesothelioma, which will shed further light on the molecular mechanisms of mesothelioma pathogenesis and malignant transformation.

## AUTHOR CONTRIBUTIONS

**Tatsuhiro Sato:** Conceptualization (lead); data curation (lead); formal analysis (lead); funding acquisition (equal); investigation (lead); project administration (lead); visualization (lead); writing – original draft (lead); writing – review and editing (lead). **Ken Akao:** Investigation (supporting); validation (supporting). **Ayuko Sato:** Investigation (supporting); writing – review and editing (supporting). **Tohru Tsujimura:** Investigation (supporting); writing – review and editing (supporting). **Satomi Mukai:** Investigation (supporting); writing – review and editing (supporting). **Yoshitaka Sekido:** Funding acquisition (equal); project administration (supporting); resources (lead); supervision (lead); writing – review and editing (supporting).

## FUNDING INFORMATION

This work was supported by JSPS KAKENHI (Grant Numbers 22 K07162 for TS, and 22 K19472, for YS), Aichi Cancer Research Foundation, Japanese Respiratory Foundation Grant and Research Grant of the Princess Takamatsu Cancer Research Fund 22–25417.

## CONFLICT OF INTEREST STATEMENT

The authors declare that they have no competing interests.

## ETHICS STATEMENT

All the protocols for research were approved by the Aichi Cancer Center Ethics Committee.

## Supporting information


Table S1.

Figure S1.
FIGURE S2.
FIGURE S3.
FIGURE S4.

## Data Availability

Data supporting the results of this study are available from the corresponding author upon reasonable request.
