# Supplementary figures and images for: Aberrant expression of NPPB through YAP1 and TAZ activation in mesothelioma with Hippo pathway gene alterations
Source: Cancer Med. 2023 May 11;12(12):13586–98. doi: 10.1002/cam4.6056 (PMC10315720; doi:10.1002/cam4.6056)

## Slide 1
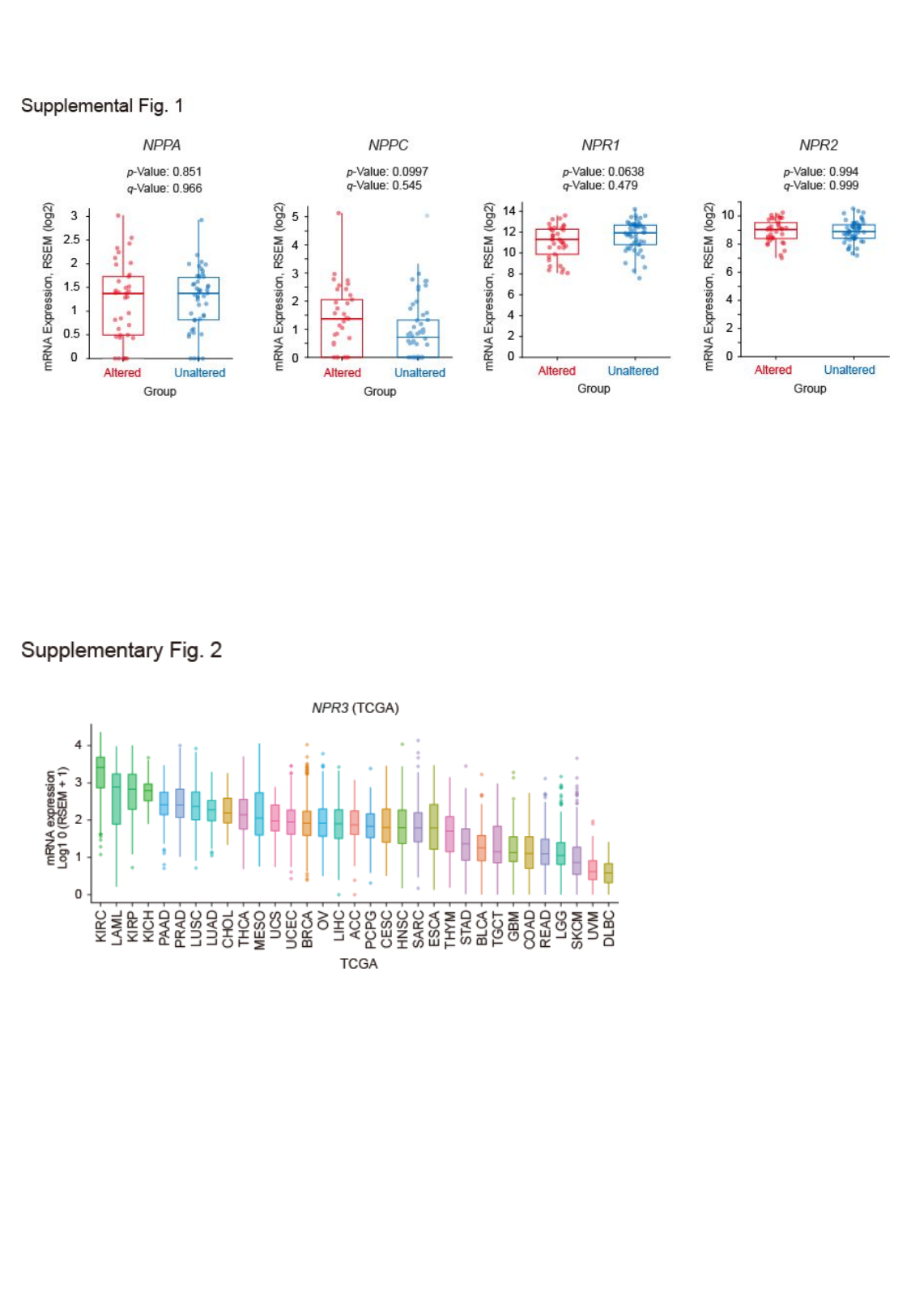

## Slide 2
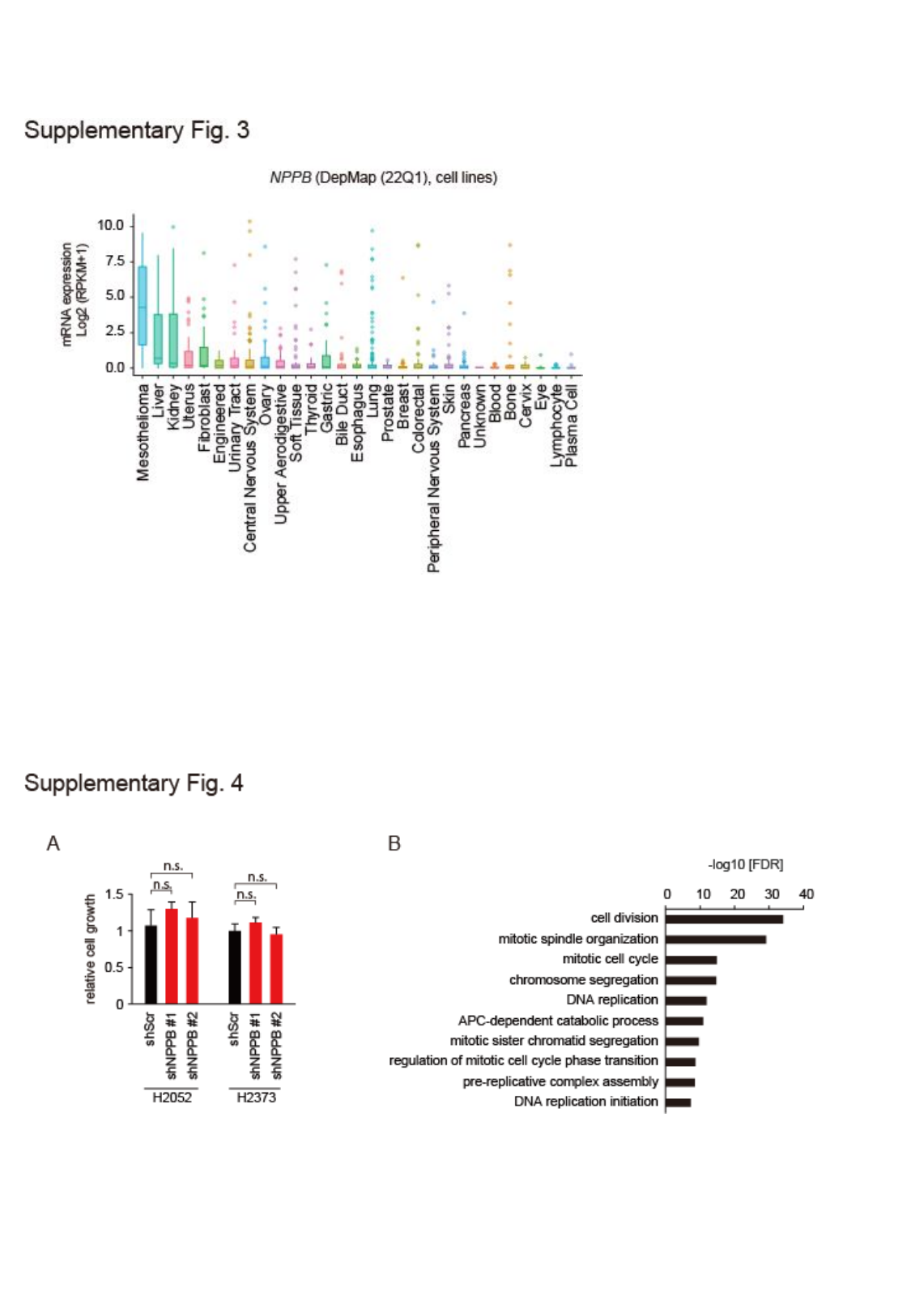

Supplement: Supplementary file 2 — Figure S1. FIGURE S2. FIGURE S3. FIGURE S4. [file CAM4-12-13586-s002.pptx]
